# Barriers to adopting and implementing an oral health programme for managing early childhood caries through primary health care providers in Lima, Peru

**DOI:** 10.1186/1472-6831-14-17

**Published:** 2014-03-06

**Authors:** Eraldo Pesaressi, Rita S Villena, Wil JM van der Sanden, Jan Mulder, Jo E Frencken

**Affiliations:** 1 Department of Paediatric Dentistry, Universidad de San Martin de Porres; Dental School, Lima, Peru; 2 Department of Global Oral Health, Radboud University Medical Center, College of Oral Sciences, P.O. Box 9101, 6500, HB, Nijmegen, the Netherlands

**Keywords:** Oral health, Early childhood caries, Caries prevention, Nurses perception, Peru, Primary health care

## Abstract

**Background:**

To identify barriers to participation in a primary oral health care programme aimed at preventing early childhood caries, as perceived by nurses.

**Methods:**

Of a total of 140 randomly selected nurses employed in 40 government health centres in Lima, 123 completed a pre-tested questionnaire. Background variables were districts’ ‘socio-economic status’ (SES) and ‘years of experience’. Factor analysis was performed. ANOVA was applied for testing the influence of the background variables on the barrier factors. Chi-square test was applied to test for differences between single item barriers and the background variables. The Likert-scale (1–4) was used.

**Results:**

There was no statistical significant effect of ‘SES’ or of ‘years of experience’ of nurses on any of the 7 barrier factors, nor on the 11 single item barrier factors. The highest mean score (3.81) was obtained for the barrier factor ‘*importance of oral health’*, followed by *‘perceived responsibility’* (3.44). The lowest mean score was (1.70) for *‘knowledge on caries prevention’*.

**Conclusions:**

Nurses consider oral health very important and are willing to participate actively in programmes aimed at reducing Early Childhood Caries, provided that they will be trained well and that the director and dentists of the health centre give their consent.

## Background

Worldwide, Early Childhood Caries (ECC) is a serious public health problem that affects, in particular, children from low-income and disadvantaged communities [1-3]. Untreated ECC can lead to serious adverse conditions affecting the psychological [4,5], social [6] and physical [7] development of children. Current care is often based on behavioural management in conjunction with invasive restorative interventions that sometimes require sedation or general anaesthesia [5,8]. This approach does not guarantee acceptable clinical outcomes, nor is it considered effective in preventing the occurrence of new caries lesions. Furthermore, treatment under general anaesthesia is expensive and risky.

ECC is preventable [9,10]. It has been suggested that providing preventive oral care for children at risk of ECC within the first year of life is crucial [11,12], as is providing oral health education [13]. However, dental visits during early childhood are infrequent, owing to a number of factors that are country and culture dependent [14]. In many countries infants and toddlers regularly visit health centres for vaccinations and well-child controls and advice, delivered by Primary Health Care Providers (PHCP) [15,16]. PHCPs, in most cases, have not been trained in oral healthcare and they do not counsel young children or parents about the prevention of ECC [17,18]. However, if trained, they could educate parents and/or caretakers about good oral health behaviour and about detecting early signs of ECC.

In Peru, ECC is a public health problem. Its prevalence among 0-11, 12-23, 24-35 and 36-47 months-old infants from deprived areas of Lima (capital of Peru) is 10.5%, 27.3%, 60% and 65.5%, respectively [19]. This high prevalence needs a multi-disciplinary approach. Collaboration with PHCPs employed in the Peruvian Public Health Framework, such as nurses, would be advantageous. Nurses see mothers during pregnancy and after birth, at 3- or 5-month intervals, when their infants require immunization and well-child controls. Hence, adding good oral health education and maintenance activities to the duties of nurses would provide a huge potential for sustaining healthy dentition and reversing the current ECC situation. However, for such duties to become effective, possible barriers to adoption and implementation by nurses of the required oral healthcare activities need first to be addressed [20,21].

The aim of the present study was to identify the barriers that nurses in Lima, Peru could experience in adopting and implementing a primary oral healthcare programme targeted at infants and their caretakers in order to prevent early childhood caries.

## Methods

### Development of a questionnaire

A validated questionnaire identifying barriers to the adoption and implementation of a preventive oral health programme for use in the Peruvian healthcare system was not available. Therefore, an appropriate questionnaire needed to be developed and validated. This process was begun by obtaining information from the literature [8,14,15,18,21]. This was summarized into statements by a team of experts from the Peruvian Association of Dentistry for Infants (ASPOB) and from the Department of Global Oral Health of the Radboud University Nijmegen, The Netherlands. Five nurses from three randomly selected health centres of the Ministry of Health (MINSA), were interviewed, in order to gain understanding about the health organization, delivery of care and constraints experienced in their daily routine, and subsequently develop a structured questionnaire. The results were used in designing an open-ended questionnaire. It was then presented to a focus group of twenty-five nurses, not previously interviewed, for discussion about the completeness and comprehensiveness of the statements. The outcomes were then discussed by the principle investigators and a support team (8 members from ASPOB). This discussion led to the construction of a closed-ended questionnaire covering the following issues: ‘Importance of oral health’, ‘perceived responsibility’, ‘intention to give advice’, ‘training’, ‘social norms’, ‘experience in seeing carious lesions’ and ‘knowledge on caries prevention’. The final questionnaire in English consisted of 34 statements, each accompanied by a 4-point Likert’s scale (1 = Totally disagree, 2 = Disagree, 3 = Agree and 4 = Totally agree).

### Closed-ended questionnaire designing process

**Figure 1:**
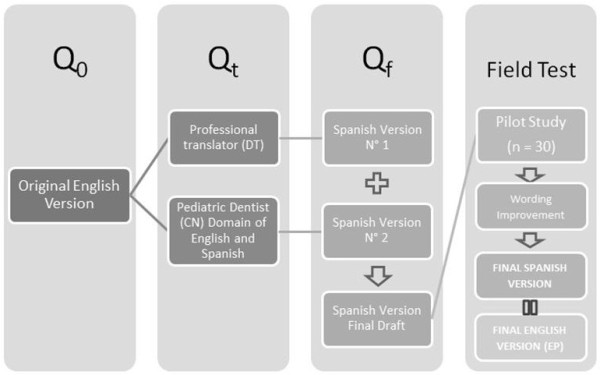
**Translation protocol to adapt the English version to a Spanish Final Version (Q**_**0 **_**= Open-ended questionnaires; Qt = Closed-ended questionnaire for test; Qf = Final questionnaire draft construction).**

After final approval was reached, the questionnaire was sent to a professional translator and to a Spanish-speaking dentist whose native language was English. Both translations were evaluated by the Spanish-speaking authors, who adjusted statements when necessary. The Spanish questionnaire was then piloted among 30 nurses who had not participated in designing the final sample. This led to improvement in the wording of three questions. The final version (Additional file 1) was re-tested among 10 nurses and once approved, was translated back to English (Additional file 2) by one of the researchers (Figure 1).

#### Sample size and randomization

In the 20 districts of Lima, MINSA operated in 554 health centres where 5,180 nurses were employed in 2012 [22]. Only health centres that had: 1) a vaccination and “well-child visit” section and; 2) followed preventive public strategies (vaccination, health education, pregnancy controls, well-child visits) were suitable for inclusion in the study. Adhering to these criteria resulted in the potential eligibility of 1,036 nurses, employed in 181 health centres. Based on an initially required sample size of 10% of nurses, and a correction factor of 10% for nurses who would have left the services by the time they would have received the questionnaire, and for a non-response of 20%, a sample size of 138 nurses was calculated.

Randomization was done as follows. The 181 health centres together with the number of nurses employed, were listed by district. Using the software programme Easy Randomizer [23], health centres were selected until the estimated sample size was reached. This resulted in a total of 140 selected nurses, originating from 40 health centres.

### Questionnaires: delivery and collection procedure

The questionnaires were delivered to and collected from each nurse, after they had provided their consent to participate in the study and they received visits from the main researcher [EP] at their workplaces over a period of one and a half months. During the first visit, the aim of the study and the lay-out of the questionnaire were explained. Contact information including their full names, health centres and telephone numbers was registered. One week later, the questionnaire was collected. A third visit was needed, as some nurses had not yet filled in the questionnaire or had lost it before the second visit.

### Data analysis

Data were entered on an Excel sheet and analysed by a statistician using SAS version 9.0. Background variables were: the socio-economic status (SES) of the district where the nurse was employed (low, medium/high) [24], and years of experience (<5; 5–10; >10). The SES of a district in Peru is based on the average income of the inhabitants and their access to basic services. Factors were constructed and analysed for confirmation, using factor analysis for principal components with Varimax rotation. A Cronbach’s coefficient alpha of ≥0.60 was considered an acceptable level of reliability of a factor. Twenty-three statements of the questionnaire were used in constructing seven barrier factors: (1) Importance of oral health; (2) Perceived responsibility; (3) Intention to give advice; (4) Training; (5) Social norms; (6) Experience in seeing carious lesions; (7) Knowledge on caries prevention (Table 1). ANOVA was applied in testing for a possible effect of the background variables on the barrier factors. Mean and standard deviations of the 11 single item barriers were calculated and the chi-square test was applied in testing for differences between these barriers and the background variables. Statistically significant difference was set at α = 0.05.

**Table 1:** Factor barriers extracted (italics), statements from which they were derived and their internal consistency (Cronbach’s α)

**Factor barrier**	**Cronbach’s α**
*Importance of oral health*	0.53
Oral health is important	
Taking care of primary teeth is important	
Primary teeth are necessary for the health of permanent teeth	
*Perceived responsibility*	0.67
Action for controlling tooth decay is necessary	
Dentist should be assisted by other health professional in managing decayed teeth	
I should assist the dentist in managing oral health	
*Intension to give advice*	0.70
I would advise mothers about habits that are beneficial for their children	
If sufficient time I would provide advice and inspect the oral cavity	
I can contribute to improvement of children’s’ oral health	
*Training*	0.71
After training, I will examine parents of the children	
If appropriate instruments are available, I will do mouth inspection	
After training, I would include oral inspection in my routine work	
*Social norms*	0.72
I participate if the dentist in the health centre accepts it	
I participate if the director of the health centre approves it	
Dental treatment must be exclusively be performed by dentists	
*Experience in seeing carious lesions*	0.66
It is common to see children with tooth decay	
I see many children with decayed primary teeth	
During routine work, I see many children with toothache	
During routine work, I see many mothers with decayed teeth	
*Knowledge on caries prevention*	0.71
Nursing bottle should be recommended from the 6th month of life	
To sweeten the milk in the nursing bottle is good	
To sleep with the nursing bottle is harmful	
Breast feeding should not be given after the 6th month of life	

Ethical clearance was obtained from the Dental School of the *San Martin de Porres University* (Lima-Peru) institutional review board (resolution N° 252-2013-D-FO-USMP). Written consent was obtained from the nurses before interviews and delivery of the questionnaires. The data was collected anonymously, as the personal information of the respondent nurses was detachable and codified.

## Results

### Disposition of respondents

A total of 123 nurses, 120 females and 3 males, from 40 health centres completed the questionnaire, which gave a response rate of 87.9%. Eighty-five percent of nurses were employed in health centres located in low, and 15% in midium/high, socio-economic areas. Twenty-two percent of nurses had less than 5 years of work experience, 36% had between 5 and 10 years and 42% had more than 10 years of work experience.

### Outcomes

There was no statistically significant effect of ‘socio-economic status’ or of ‘years of experience’ of nurses on any of the 7 barrier factors (p > 0.05). Table 2 shows the mean scores and standard deviations of the 7 barrier factors. The highest mean score (3.81) was obtained for ‘importance of oral health’, and the lowest mean score (1.70) for ‘knowledge on caries prevention’.

**Table 2:** Mean scores and standard deviations (SD) of the 7 barrier factors

**Factor**	**Mean**	**SD**
Importance of oral health	3.81	0.31
Perceived responsibility	3.44	0.50
Intension to give advice	3.31	0.53
Training	3.17	0.64
Social norms	3.04	0.63
Experience in seeing carious lesions	2.84	0.55
Knowledge on caries prevention	1.70	0.69

1 = lowest; 4 = highest.

The mean and standard deviation of single-item barrier factors and test results, by background variables, is presented in Table 3. Neither ‘socio-economic status’ nor ‘years of experience’ had a statistically significant effect on any of the single-item barrier factors. High mean scores were obtained for willingness of nurses to attend a training course and provide the requested oral health activities, while the lowest mean scores were obtained for factors related to their knowledge of caries etiological factors.

**Table 3:** Mean and standard deviation (SD) of single-item barrier factors and test results (chi-square) by background variables

**Single item barrier factor**	**Mean ± SD**		**Background variables**	
			**SES**	**YOE**
			***P***	***P***
Participation in a training course on diagnosing and preventing caries lesions	3.58	0.61	0.51	0.93
Willingness to examine children orally after training	3.44	0.68	0.92	N/A*
Examining children’s mouth is one of my official tasks	3.11	0.86	0.47	0.30
Able to recognize severely decayed teeth now	3.09	0.57	0.06	0.12
Believes that breast milk may cause tooth decay	3.15	0.71	0.66	0.59
Considers eating sugary food several times a day is harmful	1.49	0.50	0.53	0.42
Thinks that children consume sugary food several time a day	2.93	0.96	0.37	0.09
Agrees that oral hygiene should start before teeth appear	2.60	1.17	0.41	0.15
Agrees that a child should visit the dentist when the first tooth appears	2.51	1.04	0.95	0.70
Considers cavities in primary teeth acceptable because they will be replaced	2.18	0.91	0.10	0.71
Knows that it is common for children to sleep with a bottle in the mouth	1.97	0.83	0.82	0.64

*N/A = Unreliable Chi-Square test result because 22% of cells are below 5.

*SES* socio-economic status; *YOE* years of experience; 1 = lowest; 4 = highest.

## Discussion

No measuring instrument for assessing barriers to adopting and implementing a primary oral health care programme aimed at infants and children was available in the literature. Therefore, such an assessment instrument had to be developed. That fact implies that the newly constructed questionnaire could not be validated. However, face and content validation was performed and the questionnaire was judged to be valid enough for use in the present study. This and the high response rate indicates a high probability that the results present a true reflection of the opinions of nurses regarding barriers to adopting and implementing a primary oral health care program aimed at infants and children. The fact that one of the factors, ‘importance of oral health’, had a low Cronbach’s α was due to the very low deviation in outcomes, as almost all nurses reported that oral health is important.

The two major barrier factors identified were ‘importance of oral health’ and ‘perceived responsibility’. The high mean scores imply that nurses consider oral health, and particularly infant oral health, very important. They perceived that they have a role to play in assisting the dentist in the health centre, in maintaining good oral health among infants and children. The next two influential factors, ‘intention to advise’ and ‘training’, also had high mean scores. Nurses were willing to give advice to parents visiting the health centre if they received proper training in preventive oral care, mouth inspection and carious lesion detection. They will perform these duties only if there is consent from the health centre director and the dentist. The mean scores of the barrier factor ‘social norms’ clearly indicate that. Therefore, prior to development of the training course, a meeting needs to be arranged with the resident dentist(s) and health centre director, for discussion of these issues and approval of the ultimate collaborative goal of the oral health programme. Considering the strategic position of nurses, in terms of regular close contact with mothers and their infants at the health centre, they may become an important link in good oral health maintenance of under-served populations.

The fact that many nurses have seen the problem of tooth decay in children and pregnant women (‘experience in seeing carious lesions’) will add to their understanding and motivation to assist the children and if needed, adults, in maintaining and obtaining good functioning dentition. ‘Knowledge on caries prevention’ was the barrier factor with the lowest mean score. That finding is a stepping stone towards organization of a training course on oral health for nurses. Such a training course would prepare the nurses for active participation in the oral health programme and, if the dentist and director of the health centre agree to its implementation, nurses would become an important sector of the workforce helping to keep teeth of infants carious lesion-free. However, the implementation of a training program needs to be carefully planned, to guarantee the long-term adoption of the new approach. O'Brien et al. [25] reported that six out of seven studies using didactic presentations did not improve the behaviour of primary care providers, whereas seven out of eight studies covering interactive workshops reported significant subsequent improvements in professional behaviour. The findings regarding the single-item barrier factors are in line with those of the constructed barrier factors.

Traditional barriers to introduction of new methods in health education and oral health strategies in the field include time constraints, inclusion of dental procedures [16], lack of knowledge [8], and confidence related to the proposed activities [18]. Therefore, lack of knowledge and lack of self-confidence are crucial factors to be addressed before implementing oral health programmes delivered in conjunction with other health professionals [15]. Whilst there are numerous studies on reducing ECC though task-based integration of oral health aspects into existing primary healthcare structures, to our knowledge, the literature does not contain studies regarding barriers perceived by nurses, which makes a comparison of the results found in the present study with those of others not possible.

## Conclusion

Public health nurses in Lima consider oral health very important and they were very willing to participate actively in oral health programmes aimed at reducing Early Childhood Caries, provided that the directors and dentists of the health centres have given their consent and that they have been trained well in the tasks which they are supposed to perform.

## Competing interests

The authors declare that they have no competing interests.

## Authors’ contributions

EP, RV, WJMVDS and JF conceptualized and designed the study, EP collected the data, JM and JF analysed the data, EP, RV and JF drafted the manuscript. All authors read and approved the final manuscript.

## Pre-publication history

The pre-publication history for this paper can be accessed here:

http://www.biomedcentral.com/1472-6831/14/17/prepub

## Supplementary Material

Additional file 1 Spanish final version of the questionnaire used in the study. Click here for file

Additional file 2 English translation of the questionnaire final version. Click here for file
