# Supplementary material for: Barriers to adopting and implementing an oral health programme for managing early childhood caries through primary health care providers in Lima, Peru
Source: BMC Oral Health. 2014 Mar 6;14:17. doi: 10.1186/1472-6831-14-17 (PMC4016564; doi:10.1186/1472-6831-14-17)
Supplement: Additional file 1 — Spanish final version of the questionnaire used in the study. [file 1472-6831-14-17-S1.doc]

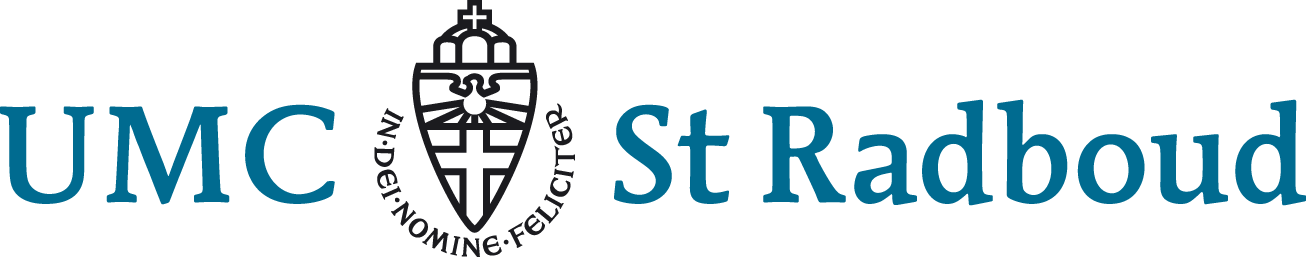

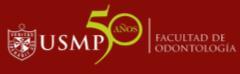
INTRODUCCIÓN

La caries dental es una enfermedad de comportamiento que parece prevalecer entre diferentes grupos de edades en el Perú. Por ejemplo, más del 50% de niños de 3 años de edad se ven afectados por esta condición. Para reducir este problema se deben buscar acciones preventivas apropiadas para detener el inicio y progresión de la enfermedad. Los odontólogos, normalmente, no atienden a niños pequeños y son las enfermeras son las que se encargan del cuidado de la salud general. Por lo tanto, las enfermeras, un profesional de la salud esencial en el cuidado de los recién nacidos, puede ser un profesional adecuado(a) para asistir a los dentistas en su lucha contra la caries dental en el Perú.

El siguiente cuestionario es el primer paso de un proyecto para promover la salud oral que busca mejorar la salud oral de los niños pequeños. Este proyecto ha sido desarrollado en conjunto por el de la Universidad Radboud de Nijmegen (Holanda) y la Universidad San Martin de Porres (Perú). Sus respuestas nos darán información útil acerca de las funciones de las enfermeras, que laboran en Centros de Salud del Ministerio de Salud del Perú (MINSA), para formular acciones preventivas que puedan ser utilizadas para reducir la prevalencia de la caries dental en los niños del Perú.

Para responder el siguiente cuestionario necesitará aproximadamente 15 minutos. Le pedimos que lea cada pregunta cuidadosamente y las responda de la mejor manera posible. El siguiente documento es completamente anónimo por lo tanto siéntase seguro de expresar su más sincera opinión. No hay respuestas correctas o incorrectas; estamos interesados en su opinión personal.

**INSTRUCCIONES**

Las preguntas en esta encuesta utilizan escalas de calificación con 4 opciones; marque con **una “X”** la respuesta que mejor describa **su** opinión. Por ejemplo, si se le pidiera calificar la frase “La comida peruana es excelente” en dicha escala, las 4 opciones se interpretarían de la siguiente manera:

|  | La comida peruana es excelente. | Totalmente en desacuerdo | Desacuerdo | De acuerdo | Totalmente de acuerdo |
| --- | --- | --- | --- | --- | --- |

Si usted es de la opinión que la Comida Peruana es **excelente**, entonces deberá marcar *‘Totalmente de* acuerdo’ de la siguiente manera:

|  | La comida peruana es excelente. | Totalmente en desacuerdo | Desacuerdo | De acuerdo | Totalmente de acuerdo |
| --- | --- | --- | --- | --- | --- |

Si usted es de la opinión que la Comida Peruana **no es muy buena**, entonces deberá marcar *‘Desacuerdo*’ de la siguiente manera:

|  | La comida peruana es excelente. | Totalmente en desacuerdo | Desacuerdo | De acuerdo | Totalmente de acuerdo |
| --- | --- | --- | --- | --- | --- |

Si usted es de la opinión que la Comida Peruana es **buena**, entonces deberá marcar *‘De acuerdo*’ de la siguiente manera:

|  | La comida peruana es excelente. | Totalmente en desacuerdo | Desacuerdo | De acuerdo | Totalmente de acuerdo |
| --- | --- | --- | --- | --- | --- |

Al realizar las calificaciones tenga en cuenta los siguientes puntos:

**Asegúrese de contestar todas las preguntas – no omita ninguna.**

**Nunca marque más de una opción por pregunta.**

**Nosotros no calificamos el cuestionario. Los números representan su opinión.**

Le pedimos que LEA cada pregunta cuidadosamente y se asegure que contestar TODAS las preguntas marcando la respuesta que mejor describe su opinión. Algunas de las preguntas pueden parecer similares, pero tratan temas distintos. No hay respuesta correcta o incorrecta. En caso de necesitar una explicación no dude en pedirla.

Este cuestionario es anónimo y todas las respuestas son confidenciales; solo pedimos un poco de información general al inicio del mismo.

Contestar las siguientes 5 preguntas de información y las 28 declaraciones.

|  | Género (marcar la opción correcta): | Femenino | Masculino |  |  |
| --- | --- | --- | --- | --- | --- |
|  |  |  |  |  |  |
|  | Edad (llenar): | . . . . | años |  |  |
|  |  |  |  |  |  |
|  | Año de graduación (llenar): | . . . . |  |  |  |
|  |  |  |  |  |  |
|  | Centro de Salud (Colocar el nombre): | . . . . |  |  |  |
|  |  |  |  |  |  |
|  | Distrito (colocar el nombre del distrito donde está ubicado el C.S.): | . . . . |  |  |  |
|  |  |  |  |  |  |
| 1 | En mi opinión, la salud oral en importante. | Totalmente en desacuerdo | Desacuerdo | De acuerdo | Totalmente de acuerdo |
|  |  |  |  |  |  |
| 2 | En mi opinión, cuidar de los dientes primarios (o de leche) es importante. | Totalmente en desacuerdo | Desacuerdo | De acuerdo | Totalmente de acuerdo |
|  |  |  |  |  |  |
| 3 | En mi opinión, los dientes primarios son necesarios para la salud de la dentadura permanente. | Totalmente en desacuerdo | Desacuerdo | De acuerdo | Totalmente de acuerdo |
|  |  |  |  |  |  |
| 4 | En mi opinión, las caries en los dientes de leche es algo aceptable porque serán reemplazados por los dientes permanentes. | Totalmente en desacuerdo | Desacuerdo | De acuerdo | Totalmente de acuerdo |
|  |  |  |  |  |  |
| 5 | En mi opinión, ingerir alimentos con azúcar varias veces al día es dañino para los dientes de leche. | Totalmente en desacuerdo | Desacuerdo | De acuerdo | Totalmente de acuerdo |
|  |  |  |  |  |  |
| 6 | Ver dentro de las bocas de los niños es una de mis actividades en el Programa “Crecimiento y Desarrollo” de MINSA. | Totalmente en desacuerdo | Desacuerdo | De acuerdo | Totalmente de acuerdo |
|  |  |  |  |  |  |
| 7 | Son necesarias acciones de salud oral para controlar las caries en los niños. | Totalmente en desacuerdo | Desacuerdo | De acuerdo | Totalmente de acuerdo |
|  |  |  |  |  |  |
| 8 | En el centro de salud es común ver a niños con dientes cariados. | Totalmente en desacuerdo | Desacuerdo | De acuerdo | Totalmente de acuerdo |
|  |  |  |  |  |  |
| 9 | Veo muchos niños con dientes de leche cariados. | Totalmente en desacuerdo | Desacuerdo | De acuerdo | Totalmente de acuerdo |
| 10 | Como enfermera en un centro de salud, sería capaz de reconocer dientes (severamente) cariados en los niños. | Totalmente en desacuerdo | Desacuerdo | De acuerdo | Totalmente de acuerdo |
|  |  |  |  |  |  |
| 11 | Otros profesionales del cuidado de la salud deberían ayudar al dentista en el manejo de dientes cariados. | Totalmente en desacuerdo | Desacuerdo | De acuerdo | Totalmente de acuerdo |
|  |  |  |  |  |  |
| 12 | Como enfermera en un centro de salud, debería ayudar al dentista en el cuidado de la salud bucal. | Totalmente en desacuerdo | Desacuerdo | De acuerdo | Totalmente de acuerdo |
|  |  |  |  |  |  |
| 13 | Participaré en un curso de capacitación sobre prevención de la caries y diagnóstico de caries para niños organizados por el departamento de salud local. | Totalmente en desacuerdo | Desacuerdo | De acuerdo | Totalmente de acuerdo |
|  |  |  |  |  |  |
| 14 | Realizaré un examen oral en los niños en el centro de salud después de haber recibido capacitación para ello. | Totalmente en desacuerdo | Desacuerdo | De acuerdo | Totalmente de acuerdo |
|  |  |  |  |  |  |
| 15 | Realizaré un examen oral a los padres de un niño en el centro de salud si estoy capacitada para hacerlo. | Totalmente en desacuerdo | Desacuerdo | De acuerdo | Totalmente de acuerdo |
|  |  |  |  |  |  |
| 16 | Miraré en la boca de los niños si tengo los instrumentos adecuados para realizar la tarea. | Totalmente en desacuerdo | Desacuerdo | De acuerdo | Totalmente de acuerdo |
|  |  |  |  |  |  |
| 17 | Participaré en el programa de salud oral, si el dentista del centro de salud estuviera de acuerdo. | Totalmente en desacuerdo | Desacuerdo | De acuerdo | Totalmente de acuerdo |
|  |  |  |  |  |  |
| 18 | Participaré en el programa de salud oral, si el Director de Salud del centro está plenamente informado acerca del mismo y lo aprueba. | Totalmente en desacuerdo | Desacuerdo | De acuerdo | Totalmente de acuerdo |
|  |  |  |  |  |  |
| 19 | Asesoraría a las madres sobre los hábitos para la buena salud oral de su hijo. | Totalmente en desacuerdo | Desacuerdo | De acuerdo | Totalmente de acuerdo |
|  |  |  |  |  |  |
| 20 | Realizaría exámenes orales y daría consejos sobre la salud oral sólo si tengo tiempo suficiente para ello. | Totalmente en desacuerdo | Desacuerdo | De acuerdo | Totalmente de acuerdo |
|  |  |  |  |  |  |
| 21 | En mi opinión, puedo contribuir a mejorar la salud oral de los niños mediante la realización de un examen oral. | Totalmente en desacuerdo | Desacuerdo | De acuerdo | Totalmente de acuerdo |
|  |  |  |  |  |  |
| 22 | Después de recibir una capacitación adecuada, cambiaré mi trabajo rutinario de las visitas a niños e incluiré inspecciones orales a los infantes. | Totalmente en desacuerdo | Desacuerdo | De acuerdo | Totalmente de acuerdo |
| 23 | Después de recibir una capacitación adecuada, cambiaré mi trabajo rutinario de las visitas a niños e incluiré inspecciones orales de los infantes. | Totalmente en desacuerdo | Desacuerdo | De acuerdo | Totalmente de acuerdo |
|  |  |  |  |  |  |
| 24 | Los tratamientos y cuidados de salud oral deben ser realizados exclusivamente por el dentista. | Totalmente en desacuerdo | Desacuerdo | De acuerdo | Totalmente de acuerdo |
|  |  |  |  |  |  |
| 25 | La lactancia materna debe ser exclusiva hasta los 6 meses de edad. | Totalmente en desacuerdo | Desacuerdo | De acuerdo | Totalmente de acuerdo |
|  |  |  |  |  |  |
| 26 | La leche, si no se elimina completamente de la boca, podría ocasionar caries. | Totalmente en desacuerdo | Desacuerdo | De acuerdo | Totalmente de acuerdo |
|  |  |  |  |  |  |
| 27 | Debe recomendarse el uso del biberón desde el sexto mes de vida. | Totalmente en desacuerdo | Desacuerdo | De acuerdo | Totalmente de acuerdo |
|  |  |  |  |  |  |
| 28 | Recomiendo endulzar la leche del biberón con azúcar. | Totalmente en desacuerdo | Desacuerdo | De acuerdo | Totalmente de acuerdo |
|  |  |  |  |  |  |
| 29 | Dormir con el biberón es una costumbre común entre las familias que asisten al centro. | Totalmente en desacuerdo | Desacuerdo | De acuerdo | Totalmente de acuerdo |
|  |  |  |  |  |  |
| 30 | Dormir con el biberón puede dañar la salud oral del niño. | Totalmente en desacuerdo | Desacuerdo | De acuerdo | Totalmente de acuerdo |
|  |  |  |  |  |  |
| 31 | La primera visita al dentista debe hacerse apenas aparezca el primer diente. | Totalmente en desacuerdo | Desacuerdo | De acuerdo | Totalmente de acuerdo |
|  |  |  |  |  |  |
| 32 | En mi opinión, consumir alimentos azucarados varias veces al día es común en los niños locales. | Totalmente en desacuerdo | Desacuerdo | De acuerdo | Totalmente de acuerdo |
|  |  |  |  |  |  |
| 33 | Durante el trabajo de rutina, veo a muchos niños que sufren de dolor de dientes. | Totalmente en desacuerdo | Desacuerdo | De acuerdo | Totalmente de acuerdo |
|  |  |  |  |  |  |
| 34 | Durante el trabajo de rutina, veo a muchas madres jóvenes con dientes cariados. | Totalmente en desacuerdo | Desacuerdo | De acuerdo | Totalmente de acuerdo |
