# Supplementary material for: Barriers to adopting and implementing an oral health programme for managing early childhood caries through primary health care providers in Lima, Peru
Source: BMC Oral Health. 2014 Mar 6;14:17. doi: 10.1186/1472-6831-14-17 (PMC4016564; doi:10.1186/1472-6831-14-17)
Supplement: Additional file 2 — English translation of the questionnaire final version. [file 1472-6831-14-17-S2.docx]

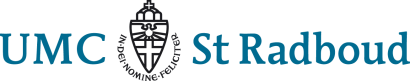

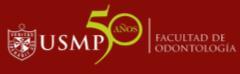
INTRODUCTION

Dental caries is a disease which seems to prevail amongst different groups of age in Peru. For instance, more than 50% of 3-year-old children are affected by dental caries. In order to reduce this problem, we have to look for appropriate preventive actions to stop the beginning of this disease. Usually, dentists do not attend small children; but nurses are those who are in charge of taking care of their integral health. Therefore, nurses, who are essential health professionals in charge of taking care of newborns, may be an appropriate ally to help dentists to fight against dental caries in Peru.

The following questionnaire is the first step of a project to encourage oral health in children. This project has been developed along with the St. Radboud University Medical Center of Nijmegen (The Netherlands) and San Martin de Porres University (Peru). Your answers will provide useful information about nurses’ duties who work in Health Centers of the Health Department of Peru (MINSA) in order to take preventive actions which may be used to reduce the prevalence of dental caries amongst Peruvian children.

In order to answer the following questionnaire you will need 15 minutes approximately. You are request to read carefully each question and answer them the best as you can. The following document is completely anonymous. Therefore, be sure that you express your most sincere opinion. There are no correct or incorrect answers. We are interested in your personal opinion.

**INSTRUCTIONS**

The questions in this survey use a marking scale with 4 options. **Cross** the answer that better describes your opinion. For instance, if you are required to assess the statement “Peruvian food is excellent” in that scale, the 4 options shall be construed as follows:

|  | Peruvian food is excellent. | I totally disagree | I disagree | I agree | I totally agree |
| --- | --- | --- | --- | --- | --- |

If in your opinion Peruvian food is **excellent**, then, you shall cross *“I totally agree”* as follows:

|  | Peruvian food is excellent. | I totally agree | I disagree | I agree | I totally agree |
| --- | --- | --- | --- | --- | --- |

If in your opinion Peruvian food **is not so good**, then, you shall cross *‘I disagree*’ as follows:

|  | Peruvian food is excellent. | I totally agree | I disagree | I agree | I totally agree |
| --- | --- | --- | --- | --- | --- |

If in your opinion Peruvian food is **good,** then, you shall cross *‘I agree’* as follows:

|  | Peruvian food is excellent. | I totally agree | I disagree | I agree | I totally agree |
| --- | --- | --- | --- | --- | --- |

Upon assessment, please take into account the following:

**Be sure that you answer all of the questions – do not omit anyone.**

**Never choose more than one option per question.**

**We do not assess the questionnaire. Numbers represent your opinion.**

**You are required to provide your opinion (last page) about topics/questions that were not completely clear for you.**

Please read carefully each question and be sure that you answer ALL of the questions, choose the answer which better describes your opinion. Some questions may seem similar but they deal with different topics. There are no correct or incorrect answers. Should you need any explanation, do not hesitate to ask for it.

This questionnaire is anonymous and all the answers are confidential. You are required only some essential information.

Answer the following 5 information questions and 34 statements.

|  | Gender (choose the correct answer): | Female | Male |  |  |
| --- | --- | --- | --- | --- | --- |
|  |  |  |  |  |  |
|  | Age (fill in): | . . . . | years |  |  |
|  |  |  |  |  |  |
|  | Graduation year at University (fill in): | . . . . | (year) |  |  |
|  |  |  |  |  |  |
|  | Health Center (Put the name): | . . . . |  |  |  |
|  |  |  |  |  |  |
|  | District (put the name of the district): | . . . . |  |  |  |
|  |  |  |  |  |  |
| 1 | In my opinion, oral health is important. | I totally disagree | I disagree | I agree | I totally agree |
|  |  |  |  |  |  |
| 2 | In my opinion, taking care of primary teeth is important. | I totally disagree | I disagree | I agree | I totally agree |
|  |  |  |  |  |  |
| 3 | In my opinion, primary teeth are necessary for permanent tooth health. | I totally disagree | I disagree | I agree | I totally agree |
|  |  |  |  |  |  |
| 4 | In my opinion, primary teeth with tooth decay are acceptable because they will be replaced by permanent teeth. | I totally disagree | I disagree | I agree | I totally agree |
|  |  |  |  |  |  |
| 5 | In my opinion, to eat sugared food several times a day is harmful for primary teeth. | I totally disagree | I disagree | I agree | I totally agree |
|  |  |  |  |  |  |
| 6 | To see inside children’s mouths is one of my activities in the “Growing and Development” Program of MINSA. | I totally disagree | I disagree | I agree | I totally agree |
|  |  |  |  |  |  |
| 7 | They are necessary health actions to control tooth decay in children. | I totally disagree | I disagree | I agree | I totally agree |
|  |  |  |  |  |  |
| 8 | It is common to see children with tooth decay. | I totally disagree | I disagree | I agree | I totally agree |
|  |  |  |  |  |  |
| 9 | I see a lot of children with decayed milk teeth. | I totally disagree | I disagree | I agree | I totally agree |
| 10 | As a nurse in a health center, I would be able to recognize teeth severely decayed in children. | I totally disagree | I disagree | I agree | I totally agree |
|  |  |  |  |  |  |
| 11 | Other health professionals should assist the dentist to manage decayed teeth. | I totally disagree | I disagree | I agree | I totally agree |
|  |  |  |  |  |  |
| 12 | As a nurse in a health center, I should assist the dentist to manage oral health. | I totally disagree | I disagree | I agree | I totally agree |
|  |  |  |  |  |  |
| 13 | I would participate in a training course on prevention and diagnosis of tooth decay for children organized by the local health department. | I totally disagree | I disagree | I agree | I totally agree |
|  |  |  |  |  |  |
| 14 | I would make an oral examination in children in the health center after having received training for that. | I totally disagree | I disagree | I agree | I totally agree |
|  |  |  |  |  |  |
| 15 | I would make an oral exam to parents of a child in the health center if I am trained for that. | I totally disagree | I disagree | I agree | I totally agree |
|  |  |  |  |  |  |
| 16 | I would look children’s mouth if I have the appropriate instruments to do it. | I totally disagree | I disagree | I agree | I totally agree |
|  |  |  |  |  |  |
| 17 | I would participate in the oral health program, if the dentist of the health center accepts it. | I totally disagree | I disagree | I agree | I totally agree |
|  |  |  |  |  |  |
| 18 | I would participate in the oral health program, if, the Health Director of the Center is completely informed about the same and if he/she approves it. | I totally disagree | I disagree | I agree | I totally agree |
|  |  |  |  |  |  |
| 19 | I would advise the mother about the habits for good oral health of her son. | I totally disagree | I disagree | I agree | I totally agree |
|  |  |  |  |  |  |
| 20 | I would make oral exams and would advise about oral health only if I have enough time to do it. | I totally disagree | I disagree | I agree | I totally agree |
|  |  |  |  |  |  |
| 21 | In my opinion, I can contribute to improve children’s oral health through oral examination. | I totally disagree | I disagree | I agree | I totally agree |
|  |  |  |  |  |  |
| 22 | Having received an appropriate training, I would change my routine job of visiting children and I would include oral inspections to children. | I totally disagree | I disagree | I agree | I totally agree |
|  |  |  |  |  |  |
| 23 | Treatments and oral health care must be performed exclusively by the dentist. | I totally disagree | I disagree | I agree | I totally agree |
|  |  |  |  |  |  |
| 24 | Breast-feeding must be exclusive up to 6 months of age. | I totally disagree | I disagree | I agree | I totally agree |
|  |  |  |  |  |  |
| 25 | If the milk is not completely eliminated from the mouth could cause tooth decay. | I totally disagree | I disagree | I agree | I totally agree |
|  |  |  |  |  |  |
| 26 | The use of nursing bottle must be recommended from the sixth month of life. | I totally disagree | I disagree | I agree | I totally agree |
|  |  |  |  |  |  |
| 27 | I recommend to sweet the milk of the nursing bottle. | I totally disagree | I disagree | I agree | I totally agree |
|  |  |  |  |  |  |
| 28 | To sleep with the nursing bottle is a common habit amongst families who attend to the center. | I totally disagree | I disagree | I agree | I totally agree |
|  |  |  |  |  |  |
| 29 | To sleep with the nursing bottle may be harmful for children’s oral health | I totally disagree | I disagree | I agree | I totally agree |
|  |  |  |  |  |  |
| 30 | The first visit to the dentist should be as soon as the first tooth appears. | I totally disagree | I disagree | I agree | I totally agree |
|  |  |  |  |  |  |
| 31 | Oral hygiene must should begin before the first tooth appears. | I totally disagree | I disagree | I agree | I totally agree |
|  |  |  |  |  |  |

| 32 | In my opinion, to consume sugared food several times a day is common amongst local children. | I totally disagree | I disagree | I agree | I totally agree |
| --- | --- | --- | --- | --- | --- |
|  |  |  |  |  |  |
| 33 | During the routine work, I see a lot of children who suffer from tooth pain. | I totally disagree | I disagree | I agree | I totally agree |
|  |  |  |  |  |  |
| 34 | During the routine work, I see a lot of young mothers with decayed teeth. | I totally disagree | I disagree | I agree | I totally agree |
